# Kite versus Ponseti Method in the Treatment of 235 Feet With Idiopathic Clubfoot: Results of a Single Romanian Medical Center

**DOI:** 10.1097/MD.0000000000001379

**Published:** 2015-08-21

**Authors:** Zoltan Derzsi, Örs Nagy, Horea Gozar, Simona Gurzu, Tudor Sorin Pop

**Affiliations:** From the Department of Pediatric Surgery (ZD, HG); Department of Orthopedic Surgery (ÖN, TSP); and Department of Pathology, University of Medicine and Pharmacy, Tirgu-Mures, Romania (SG).

## Abstract

Congenital idiopathic clubfoot (CC) represents the fifth common most congenital malformation which may be treated conservatively or by surgery. In this article, we present the results obtained in our clinic after conservative therapy performed with 2 methods.

A total of 235 consecutive feet (161 patients) were conservatively treated using Kite (n = 129) and Ponseti method (n = 106). The Dimeglio score was determined before and at 6 months after treatment to compare the 2 methods. All of the patients were treated in their first week of life.

CC was more frequently diagnosed in males (n = 93; 57.76%), bilaterality being seen in 45.96% of the patients (n = 74). Although before therapy the Dimeglio score was similar in both groups (*P* = 0.85), it was significantly improved in patients treated by Ponseti method (*P* = 0.005). Duration of therapy was also longer in patients from Kite versus Ponseti group (20 vs 11 weeks). Failure of orthopedic treatment was more frequent in Kite group (30.32% vs 8.49% of the patients) and the relapses rate at 6 months was also higher (35.65% vs 11.32%).

The conservative method used to treat the CC should be adapted on the patient's age and Ponseti method seems to be the most effective type of treatment used for patients treated in their first week of life. Dimeglio score can be successfully used for evaluation of these children. This is the eighth published study that compare the efficacy of Kite versus Ponseti method.

## INTRODUCTION

Congenital clubfoot (CC) is a malformation of the osteoarticular system that affects 0.64 to 6.8/1000 live newborns and refers to uni- or bilateral modifications of position of the affected feet in hindfoot varus, equinus, suppination, and forefoot adductus, with or without medial or posterior groove, cavus, or affected muscular function.^1–4^ It is the fifth most common congenital malformation after cardiovascular malformation, hypospadias, lesions of the urinary tract, and Down syndrome.^1,2,4^

CC may be treated conservatively or by surgery; several methods have been proposed over the years. Nowadays, 2 fundamental rules are used: treatment should be initiated from the first week of life, and the first line of treatment is always conservative.^1–7^ Although significant improvement can also occur after walking age, effectiveness of the treatment decreases with age.^2^ However, although several methods have been proposed along the years, there is no standard therapy for manipulation and casting of children with CC.^8^

One of the pioneers that firstly proposed conservative management was J. H. Kite in the United States in the 1930s (center of rotation of misaligned foot and fulcrum on cuboid); then, Ignacio Ponseti adjusted in the 1940 to 1950s the method proposed by Kite and conceived a new method of correction (fulcrum on head of talus), that was firstly published in 1963 but become more widely used in 1990s.^2–7^

Kite method aims to gradually correct each deformity by itself, starting with correcting the mediotarsal adduction, followed by correction of the internal rotation calcanopedal block and the calcaneal varus, and finally the equinus. To move on to correcting the next deformity, the previous one must be completely corrected. Basically short manipulating sessions of about 5 min are held for each foot, followed by femurocruropedal immobilization for about 2 weeks.^7^ Ponsetti method aims to correct the defining deformities (cavus, varus, and adduction) in the same time, whereas the equinus will be corrected lastly, by periodic weekly manipulations, followed by consecutive femurocruropedal immobilization.^9^ The 2 methods are still used in the daily practice, but most of the recent studies recommend Ponseti method.

In the Pediatric Surgery and Orthopaedics Clinic of Tirgu-Mures, Kite method was only used before 2009. After 2009, the Ponseti method was introduced and used by the same physicians’ team. The present study was conceived to retrospectively evaluate the results of the 2 techniques in a single medical center and to present the advantages and disadvantages of each of them, in our experience. The Dimeglio scoring system, firstly described in 1995, was used for evaluation. It is a summarizing system that takes into account 4 major and 4 minor criteria. The major criteria target the reducibility of the 4 malpositions, as follows: equinus, varus, adduction of the forefoot, and suppination. The grading is from 0 (reducible, almost normal) to 4 (nonreducible, very severe). Minor or secondary criteria are as follows: posterior groove, medial groove, cavus, and affected muscle function, which are all awarded 1 point each of present and 0 points of absent. Thereby a score between 0 and 20, and 0 points represents a normal foot; also, an increasing score means a greater severity. Thus, the Dimeglio scoring system distinguishes 4 categories: benign (stage I), moderate (stage II), severe (stage III), and very severe (stage IV).^10–12^

Although Ponseti method was extensively explored in the last time, in a meta-analysis performed by Matos and de Oliveira, it was mentioned that only 4 articles that compared the 2 methods have been published till 2010;^5,13,14^ another 3 were added during 2010 to May 2015.^3,15,16^ To best of our knowledge, this is the eighth study from English literature that compare the efficacy of Ponseti versus Kite method and comprises the largest number of cases.

## MATERIAL AND METHODS

### Inclusion and Exclusion Criteria

The present study was performed in the Pediatric Surgery and Orthopaedics Clinic from Emergency County Hospital of Târgu Mureş, Romania, and takes into account all consecutive children with clubfoot treated in our clinic between January 2007 and 2013; all of them began the treatment within their first week of life. The average post-therapy follow-up was 6 months. All those patients who did not comply during treatment and the aftercare, who have abandoned the therapy, or who did not agree to participate in the present study have been excluded. The head of the Clinic approved evaluation of the cases and publication of the results. Written informed consent was obtained from each parent prior to beginning any research.

### Management Protocol

In any patient, after careful clinical examination performed in first week of the life, the nonsurgical orthopedic treatment was performed, applying the aftercare protocol; the clinical evaluation of results was performed 6 months after finishing therapeutic protocol. In the worksheets, completed before and after therapy, the following informations were included: patient's age, sex, the affected side, associated malformations, and the Dimeglio score^9–11^ evaluated before and after treatment. There were no cases with other associated malformations included in the study. No history of familial clubfoot was noted.

Two groups of patients were retrospectively evaluated: one group in which Kite method was used (group A) and second group (group B) treated with Ponseti method.^2–7,14^

### Statistical Analysis

Statistical data were handling with GraphPad Prism 5. The distribution curve has been evaluated with the Kolmogorov–Smirnov test. The Mann–Whitney test was used to compare the central tendencies. To test the differential proportions, the *χ*^2^ test was performed. As a statistical significance, 0.05 interval of confidence was used for all of the tests.

## RESULTS

### Clinical–Pathological Characteristics

From 2007 to 2013, we treated 161 consecutive patients with CC. Of the 161 patients, 93 (57.76%) were boys and 68 (42.23%) were girls, with a median age of 5.20 ± 1.73 days (range between 1 and 7 days). No significant differences regarding the patient's age, sex, weight, tall, or other clinical-pathologic characteristics were noted.

Bilateral manifestation was seen in 74 of the patients (45.96%); a total of 235 affected feet were evaluated. From the 161 unilateral CC, the right leg was affected in 97 patients (60.25%).

From the 235 feet, 129 were treated with Kite method (group A) and 106 with Ponseti method (group B) (Table 1).

**TABLE 1:**
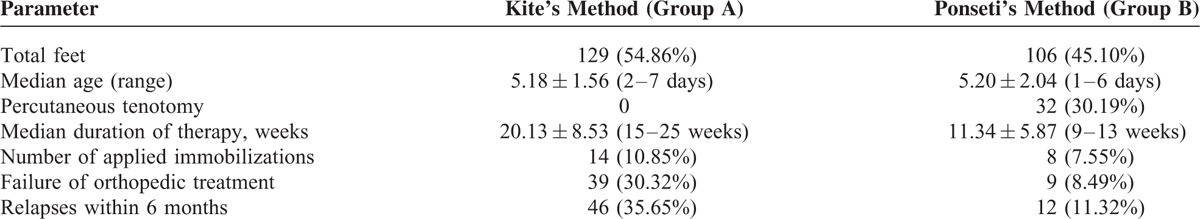
Comparison of Clinical-Pathological Characteristics of Patients With Congenital Clubfoot and Their Response at Conservative Therapy

### Comparative Results With Dimegli Score System

**FIGURE 1:**
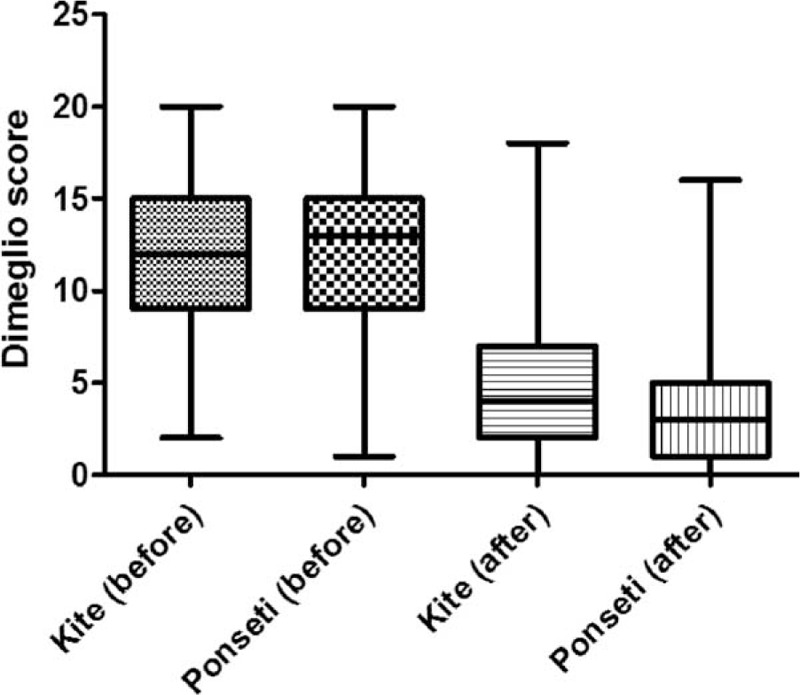
The average Dimeglio score counted before and after treatment of congenital clubfoot, using Kite (group A) and Ponseti method (group B).

**FIGURE 2:**
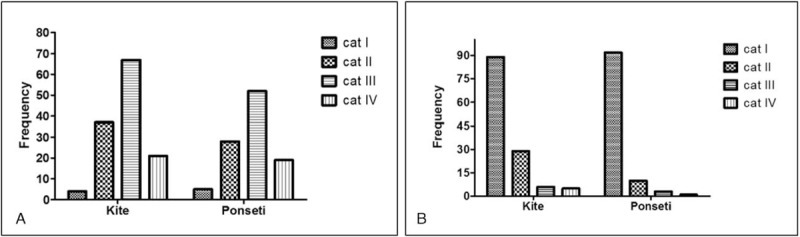
Grading of the congenital clubfoot severity, using Dimeglio score, before (A) and after treatment (B), using Kite (group A) and Ponseti method (group B).

At 6 months after therapy, the Dimeglio score (evaluated before and after therapy) showed significant differences between the 2 groups. Although the average score was similar before and after therapy in both groups (*P* = 0.87), and also stage distribution before therapy (Mann-Whitney test; *P* = 0.85), the difference was observed at distribution of the cases in the 4 stages (Mann-Whitney test; *P* = 0.005) (Table 2, Figure 1). If the post-therapy stages III and IV presented similar proportion in group A and B, a significant increased proportion of cases with stage I (chi square test; *P* = 0.01) was seen in patients from group B (Table 2, Figure 2).

**TABLE 2:**
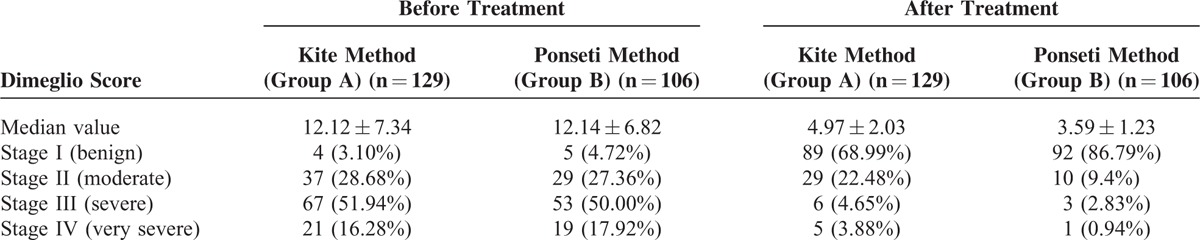
Stage Distribution of the Feet Before and After Treatment, According to the Dimeglio Scoring System

## DISCUSSION

CC can be unilateral, mostly in the right leg (60.25% of the cases in this study), but bilateral deformity was seen in 40–64% of the cases reported in literature, in line to our data.^1^ Although most of the cases are idiopathic, maternal smoking, early amniocentesis, viral infections, neurologic diseases, and patient's sex (males are more predisposed; 57.76% in this study) are considered as risk factors.^1,4^ Due to the fact that 33% of the twins present concordance and familial history was proved in 25% of the cases, involvement of the genetic factors was also supposed.^1^ The incidence of CC seems to be higher in New Zealand than in Europe but its ethnicity-related incidence is controversial.^1^

Although CC can be conservatively treated using both Kite and Ponseti method, in both before and after walking age,^2^ there are some differences among them that should be taken into account for a proper therapy. Ponseti method is mostly indicated for children treated up to 6 months to 1 year of age^2,14,17^ and our study proved that it offers superior results, compared with Kite method, till first week of life. It is worthy noticing that, in infants, not only the Dimeglio score was most significantly improved but also the 6 months’ rate of relapses was also lower in the Ponseti group, this observation being in line to other researches.^3,4^ Ponseti method superiority was also proved for the correction rate and functional outcome.^3,4^ The median success rate is 58% to 79% for Kite and 78% to 98% for Ponseti method.^14,15^ Median time of therapy is also higher using the Kite method (20 vs 10–15 weeks).^14,17^ In several studies, the superiority of Ponseti method was agreed, for both primary correction and uncorrected plus relapsed feet,^3,13^ but the risk for over-correction and stiff scar healing was higher after Ponseti than Kite method.^3^ Its superiority is also related on the lower cost and higher effectiveness^16^ and can also improve significantly the Kite recurrent clubfeet.^18^

Although Ponseti method is also used in children older than 20 months, the components of varus, medial rotation of calcaneopedal block, and adductus showed a lesser improvement than in younger children.^2^

This study proves, through a retrospective evaluation, that Ponseti method can be successfully used to correct idiopathic CC in infants, from their first week of life, with a low rate of tenotomy, and Dimeglio score can be used for their evaluation.
